# Supplementary material for: Effects of episodic slow slip on seismicity and stress near a subduction-zone megathrust
Source: Nat Commun. 2021 Dec 21;12:7253. doi: 10.1038/s41467-021-27453-8 (PMC8692312; doi:10.1038/s41467-021-27453-8)
Supplement: Supplementary file 1 — Supplementary file [file 41467_2021_27453_MOESM1_ESM.pdf]

Supplementary Materials for

Effects of episodic slow slip on seismicity and stress near a subduction-zone megathrust

Authors: Saeko Kita[1*], Heidi Houston[2], Suguru Yabe[3], Sachiko Tanaka[4], Youichi Asano[4], Takuo Shibutani[5], Naoki Suda[6]

Affiliations:

[1] Building Research Institute, National Research and Development Agency, Tsukuba, Ibaraki, Japan

[2] University of Southern California, Los Angeles, California, USA

[3] National Institute of Advanced Industrial Science and Technology (AIST), Tsukuba, Ibaraki, Japan

[4] National Institute of Earth Science and Disaster Resilience (NIED), Tsukuba, Ibaraki, Japan

[5] DPRI, Kyoto University, Uji, Kyoto, Japan

[6] Hiroshima University, Higashi-Hiroshima, Hiroshima, Japan

* Currently at the Department of Earth and Planetary Science and Berkeley Seismolab, University of California, Berkeley, California, USA

Correspondence to: kita@kenken.go.jp

This PDF file includes:

Supplementary Text 1 through 3
Supplementary Tables 1 through 4
Supplementary Figures 1 through 10
Supplementary References (1 through 4)

Supplementary Text

Supplementary Text 1

Details of stress regime for interplane and lower-plane events

Figures 2i and 2j show the results of stress tensor inversions for interplane and lower-plane events using the focal mechanisms input data shown in Figs. S2e and S2f. These results show that the direction of $\sigma1$changes by more than 40 degrees before and after SST (Figs. 2i and 2j and Supplementary Table 1). However, the 90% confidence limits on $\sigma1$ after SST are very broad, indicating difficulty distinguishing $\sigma1$ and $\sigma2$. Similarly, broad confidence limits on $\sigma1$ were also found for interplane and lower-plane events in northeastern Japan subduction zone[1], suggesting an unstable stress regime in interplane and lower-plane regions of oceanic mantle in both subduction zones.

We further divided the events inverted in Figures 2i and 2j into three regions according to downdip position: the updip of SST, under the SST zone, and downdip of the SST, and inverted those groups separately for stress orientations. For these subgroups, $\sigma1$ uncertainties are smaller than for the group of all interplane and lower-plane events, but still substantial (Supplementary Fig. 3). Changes in the stress orientations before and after SST times in regions updip and downdip of SST are possible, but these observations may mainly reflect the small number of focal mechanisms and an unstable, heterogeneous stress regime. Geological and petrological studies indicate that geofluid tends to migrate in the direction of $\sigma2$[2, 3, 4]. Since the direction of $\sigma2$ generally does not parallel to the plate interface (Supplementary Fig. 3), geofluid, if present in the oceanic mantle beneath the plate interface updip and downdip of the SST zone, can migrate up towards the plate interface.

In summary, stress changes for the interplane and lower-plane events are ambiguous, and similarly to the stress regime seen in the northeastern Japan subduction zone[1], the stress regime in the interplane and lower-plane region under Kii Peninsula appears somewhat unstable, heterogeneous, and challenging to interpret. We therefore focused most of our analysis in the main text on the upper seismic plane, which is closer to the plate interface and thus expected to respond more strongly to slow slip.

Supplementary Text 2

b-value change before and after SST times in each region

Figure 3c in the main text shows the b-values versus the assumed lower limit of the analysis magnitude (Mc) for the combined 3 regions, demonstrating that b-values are higher before the occurrence of SST. Supplementary Figures 4a through 4c show similar analyses for Regions A, B, and C, separately. Regions A and C are consistent with the overall pattern for the combined regions, whereas Region B does not show the change in b-values (red versus blue symbols). However, we note that the segmentation of tremor activity in Region B is less clear and several possible segment boundaries have been suggested, so that the association of in-slab seismicity and SST timing may be less clear. Furthermore, Region B is relatively narrow. Thus, it seems clear that b-values of in-slab earthquakes beneath Kii Peninsula generally decrease after the occurrence of SST.

Supplementary Text 3

Detailed time change of seismicity and b-values in upper-plane and other events

As shown in Fig. 3d, the b-values of in-slab events evolve with time over the SST cycle, generally peaking about 1.5 months before SST. A peak in the b-values before the SST times is also seen when in-slab events are divided into upper-plane seismicity and lower-plane and

interplane seismicity (Supplementary Figs. 5a and 5b). Furthermore, when upper-plane in-slab events are divided into those in the SST zone and those updip of the SST zone, a peak in b-values before the SST times is still seen (Supplementary Figs. 5c and 5d).

The occurrence times of in-slab events versus distance along the strike of the subducting slab is shown in Supplementary Fig. 6a and 6b. The seismicity of upper-plane events is distributed uniformly, whereas the interplane and lower-plane events occur mainly in Regions B and C.

Supplementary References (1-4):

1. Kita, S., Okada, T., Hasegawa, A., Nakajima, J., Matsuzawa, T., Existence of interplane earthquakes and neutral stress boundary between the upper and lower planes of the double seismic zone beneath Tohoku and Hokkaido, northeastern Japan. *Tectonophysics*, **496**(1), 68–82 (2010).

2. Ogawa, Y., Vrolijk, P., Control of internal structure and fluid-migration pathways within the Barbados Ridge decollement zone by strike-slip faulting: Evidence from coherence and three-dimensional seismic amplitude imaging: Discussion. *Geol. Soc. Am. Bull.* **118**, 253–254 (2006)

3. Sibson, R.H., Generation of pseudotachylyte by ancient seismic pumping. *Geophys. J. R. Astron. Soc.*, **43**, 775-789 (1975).

4. Otsubo, M., Miyakawa, A., Katayama, I., Okazaki, K., An inhomogeneous across-slab conduit controlled by intraslab stress heterogeneity in the Nankai subduction zone. *Scientific Reports*, **9**, 994 (2019).

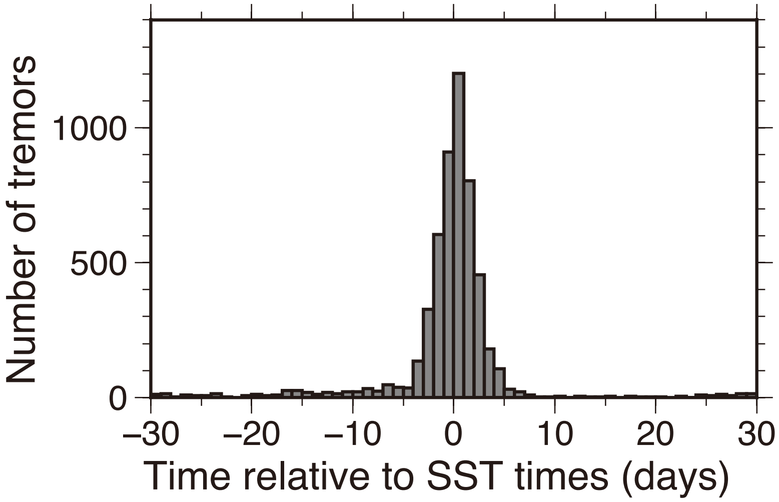

Supplementary Fig. 1.
Histogram of the relative times of all tremors within 30 days of the SST event times. The SST times were picked as the centers of major tremor bursts that span at least one segment. The histogram indicates that the typical duration of substantial tremor and inferred slip is about 8 days.

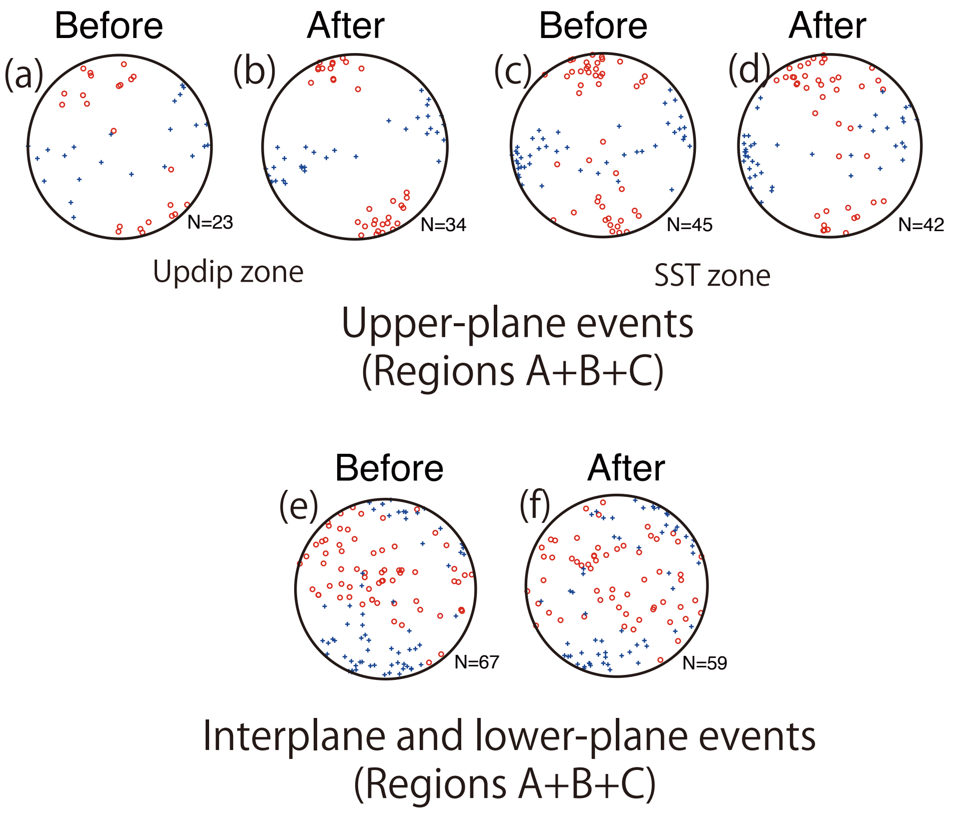

Supplementary Fig. 2.
Input data to stress tensor inversions shown in Figures 2e through 2j. (a) and (b) Input data for stress tensor inversions of upper-plane events beneath the updip of the SST zone shown in Figs. 2e and 2f, respectively. Lower hemisphere projections of the P- and T-axes, indicated by red circles and blue crosses, respectively, of focal mechanisms before and after SST times. (c) and (d) Input data for stress tensor inversions of upper-plane events beneath the SST zone shown in Figs. 2g and 2h. (e) and (f) Input data for stress tensor inversions of inter-plane and lower-plane events, respectively, shown in Figs. 2i and 2j.

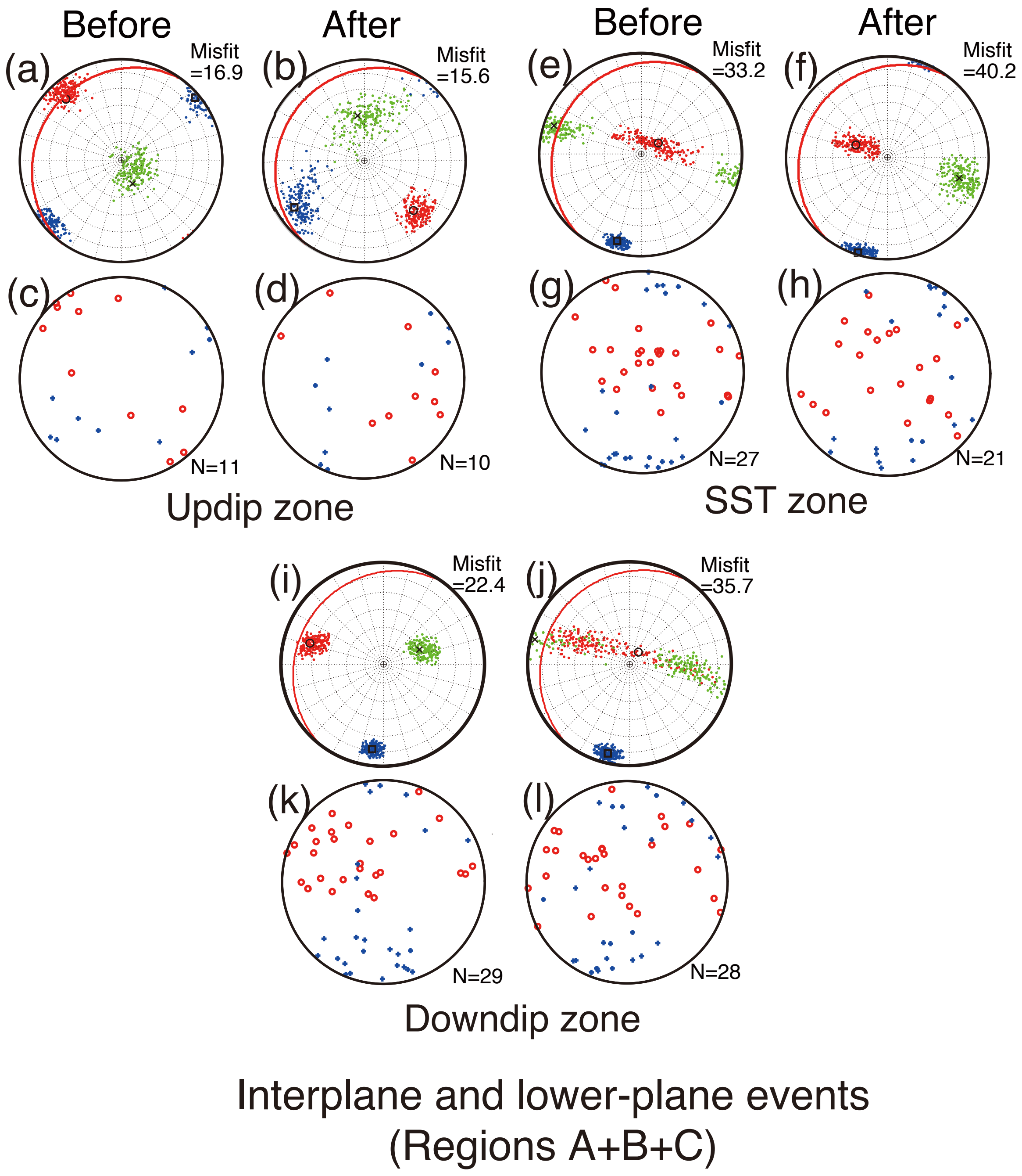

Supplementary Fig. 3.
Results of stress tensor inversions for the interplane and lower-plane events (oceanic mantle events) and input data (focal mechanisms). (a) and (b) Results for the oceanic mantle events beneath the updip of the SST zone before and after the SST times. (c) and (d) Input data for (a) and (b), respectively. (e) and (f) Results of the inversions beneath the SST zone. (g) and (h) Input data for (e) and (f), respectively. (i) and (j) Results beneath the downdip of the SST zone. (k) and (l) Input data for (i) and (j), respectively.

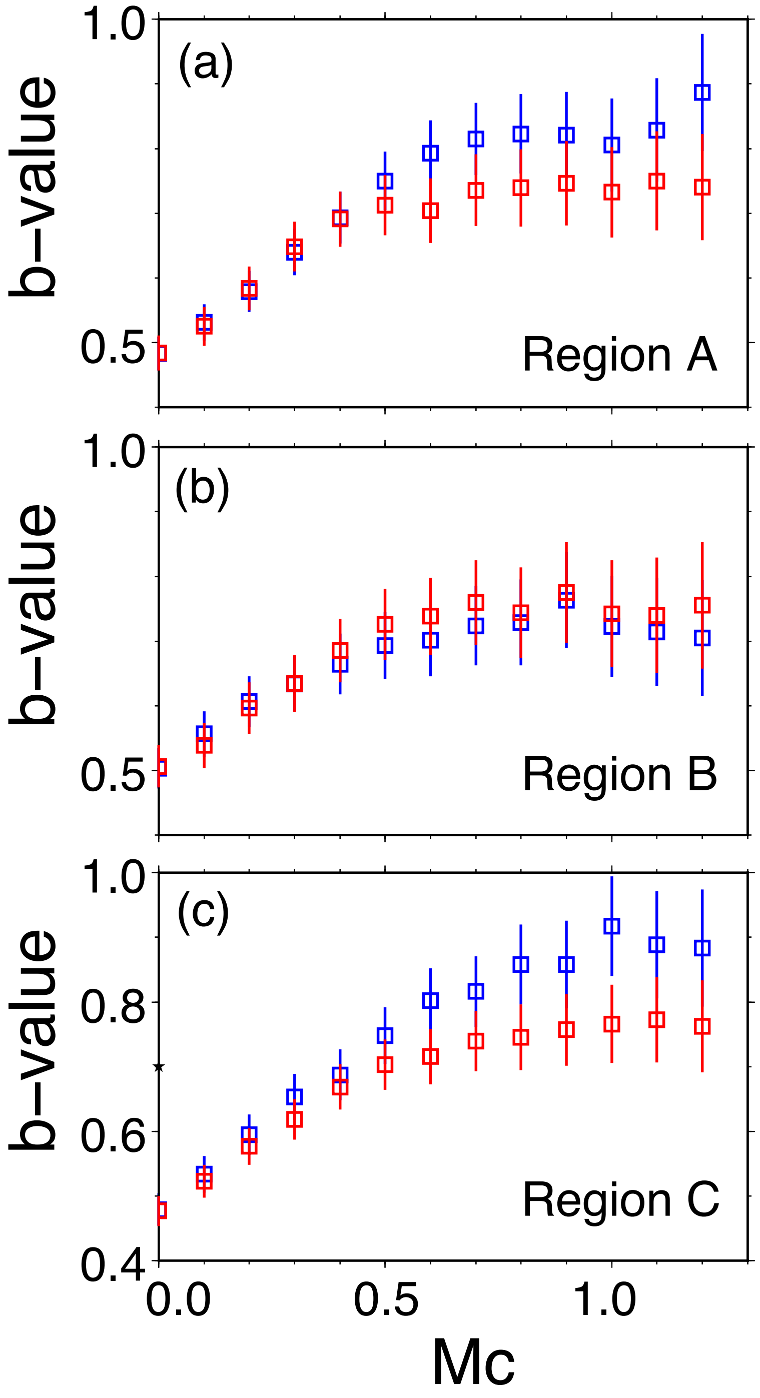

Supplementary Fig. 4.

b-value versus assumed magnitude of completeness (Mc) for (a) Region A, (b) Region B, and (c) Region C. Red dots indicate b-values for events that occurred in two-month time windows before SST times, and blue dots indicate b-values for events in two-month windows after SST times.

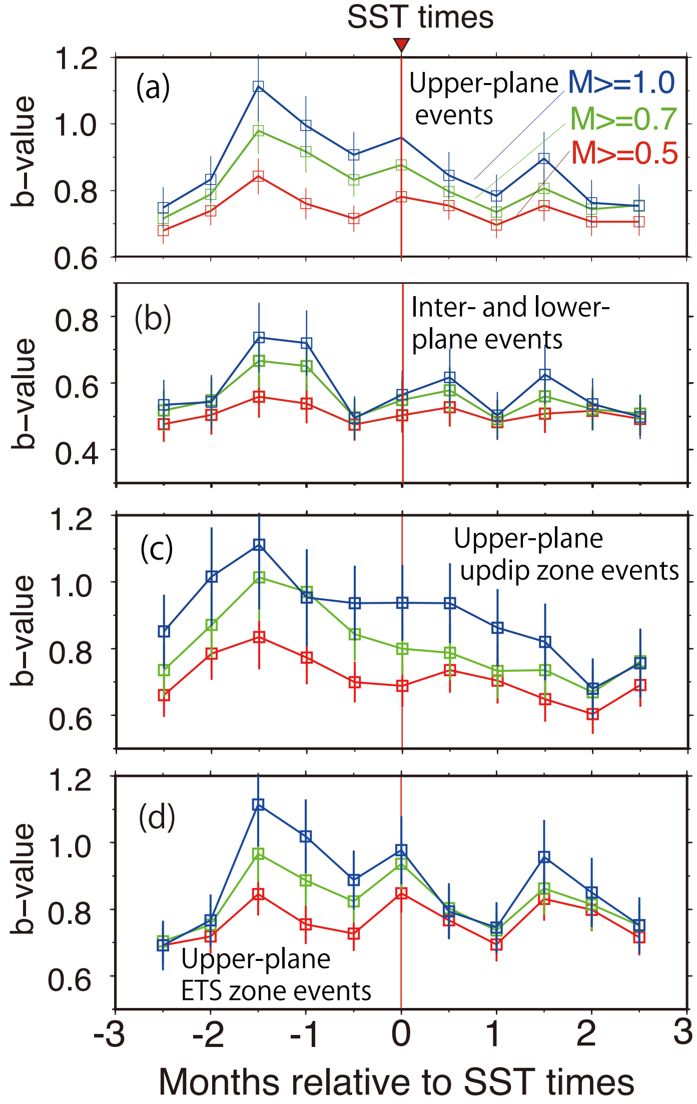

Supplementary Fig. 5.
b-value time evolution for (a) upper-plane events, (b) lower-plane and interplane events, (c) upper-plane events beneath the updip zone, and (d) upper-plane events beneath the SST zone for the combined region using one-month moving windows. Red, green, and blue dots indicate the estimated b-values for Mc values of 0.5, 0.7 and 1.0, respectively.

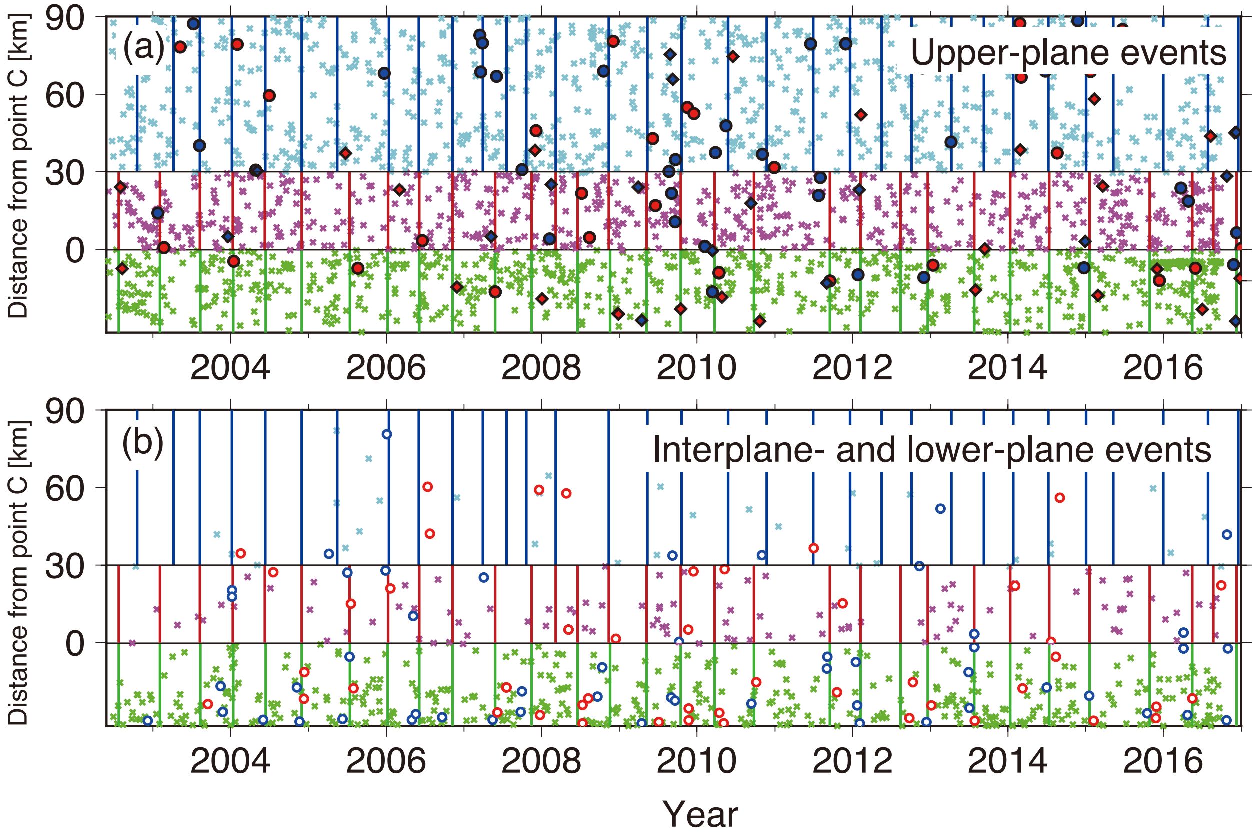

Supplementary Fig. 6.

In-slab earthquake occurrence times versus distance along the strike of the subducting slab. Gray crosses indicate (a) upper-plane events and (b) lower-plane and interplane events. The division of regions is shown in Fig. 1a. The distance along the strike is measured from point C in Fig. 1a.

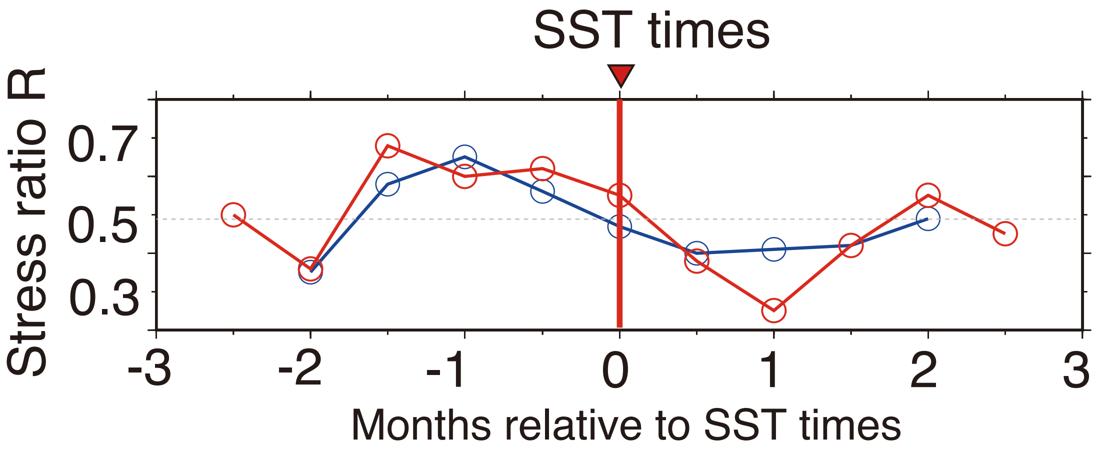

Supplementary Fig. 7.

Time evolution of stress ratio R for all in-slab events. R values for stress inversions of all in-slab events using one and two-month moving windows are indicated by red and blue dots, respectively. A value of R close to 0 represents a stress state dominated by single-axis tension, whereas a value of R close to 1 represents a stress state dominated by single-axis compression. Note that the time change of R in the present study that indicates the dominant stress regime in the entire slab changes over the SST cycle.

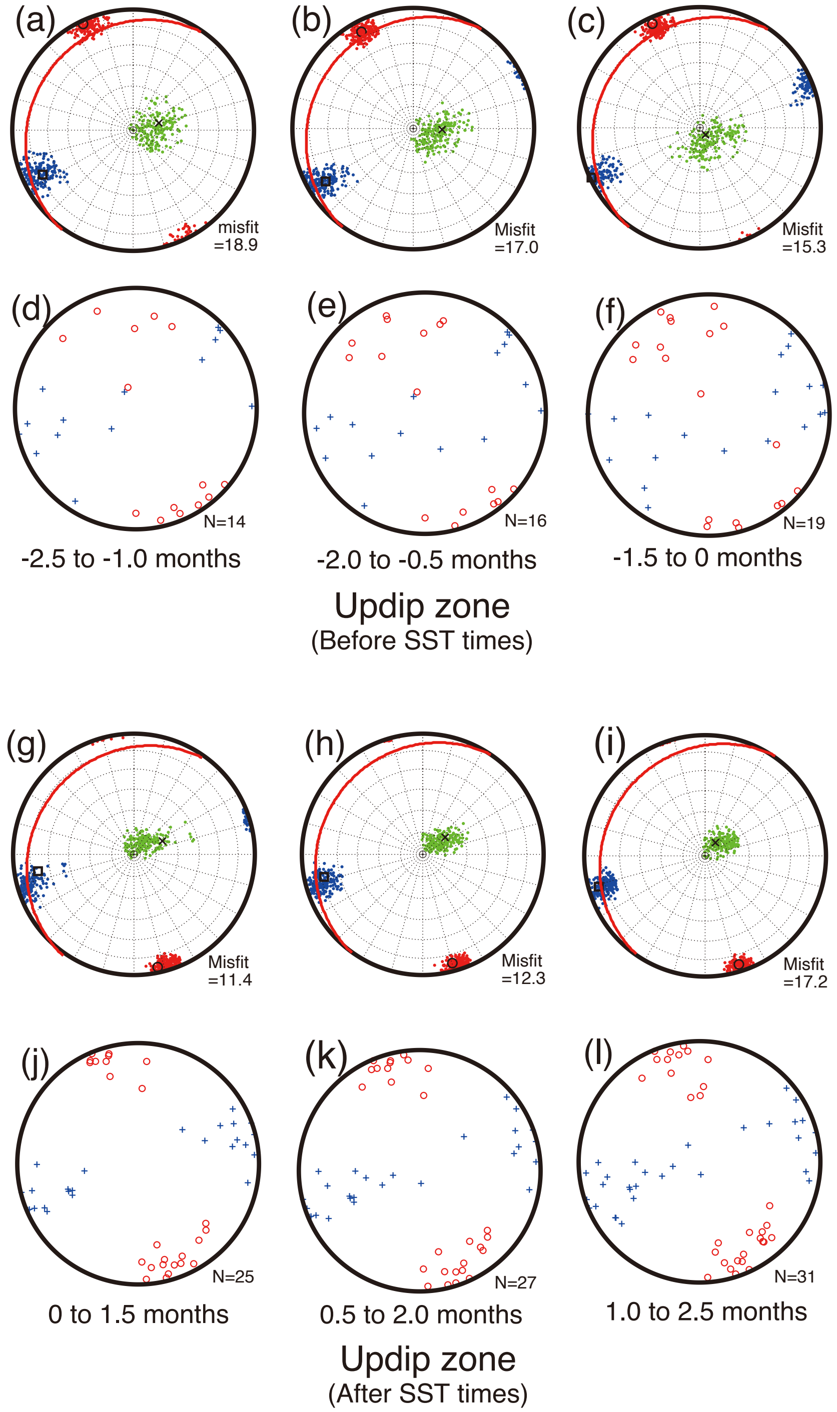

Supplementary Fig. 8.
Results of stress tensor inversions for the upper-plane events and input data (focal mechanism P- and T-axes) beneath the zone updip of SST using a 1.5-month time window.
(a), (b) and (c) Results before the SST times using the input data of (d), (e) and (f), respectively. (g), (h) and (i) Results after the SST times using the input data of (j), (k) and (l), respectively.

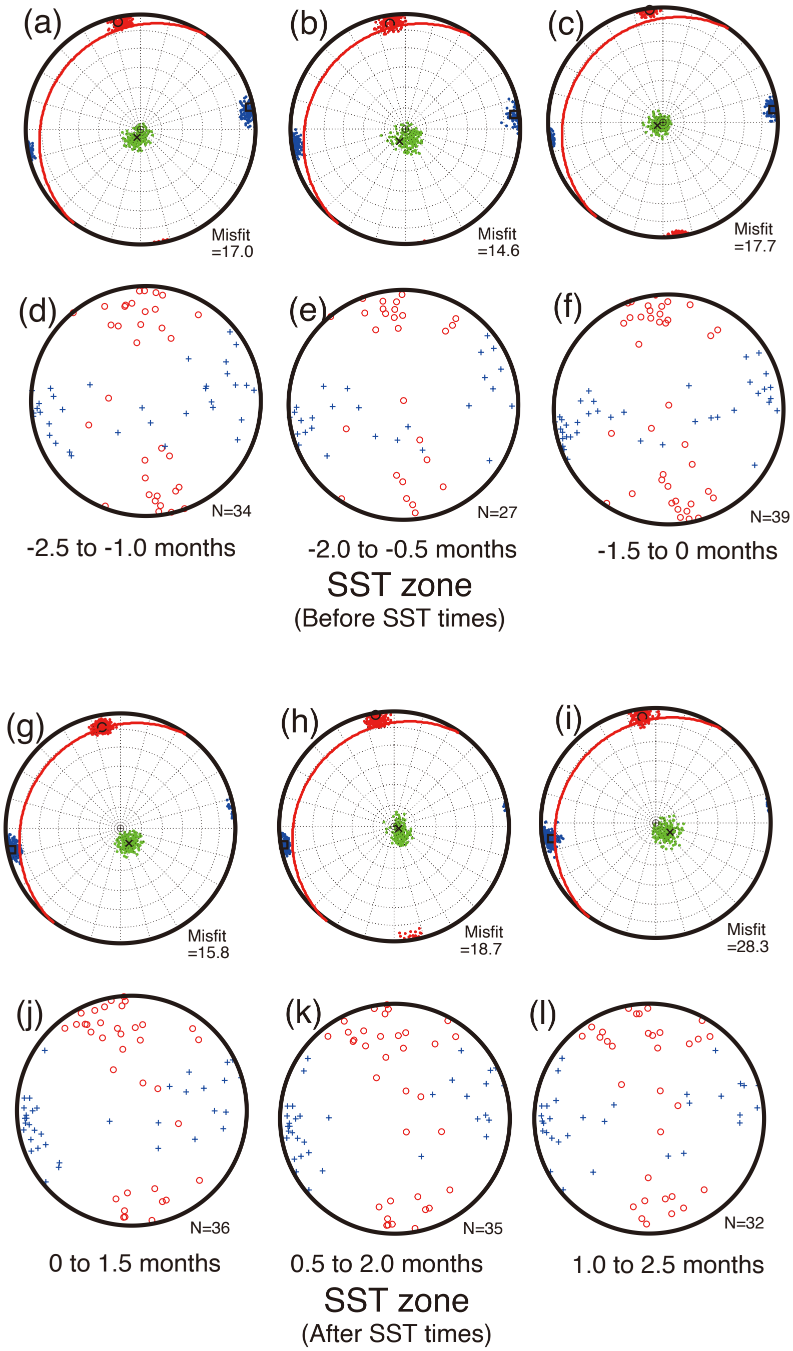

Supplementary Fig. 9.
Results of stress tensor inversions for the upper-plane events and input data (focal mechanism P- and T-axes) beneath the SST zone using a 1.5-month time window. (a), (b) and (c) Results before the SST times using the input data of (d), (e) and (f), respectively. (g), (h) and (i) Results after the SST times using the input data of (j), (k) and (l), respectively.

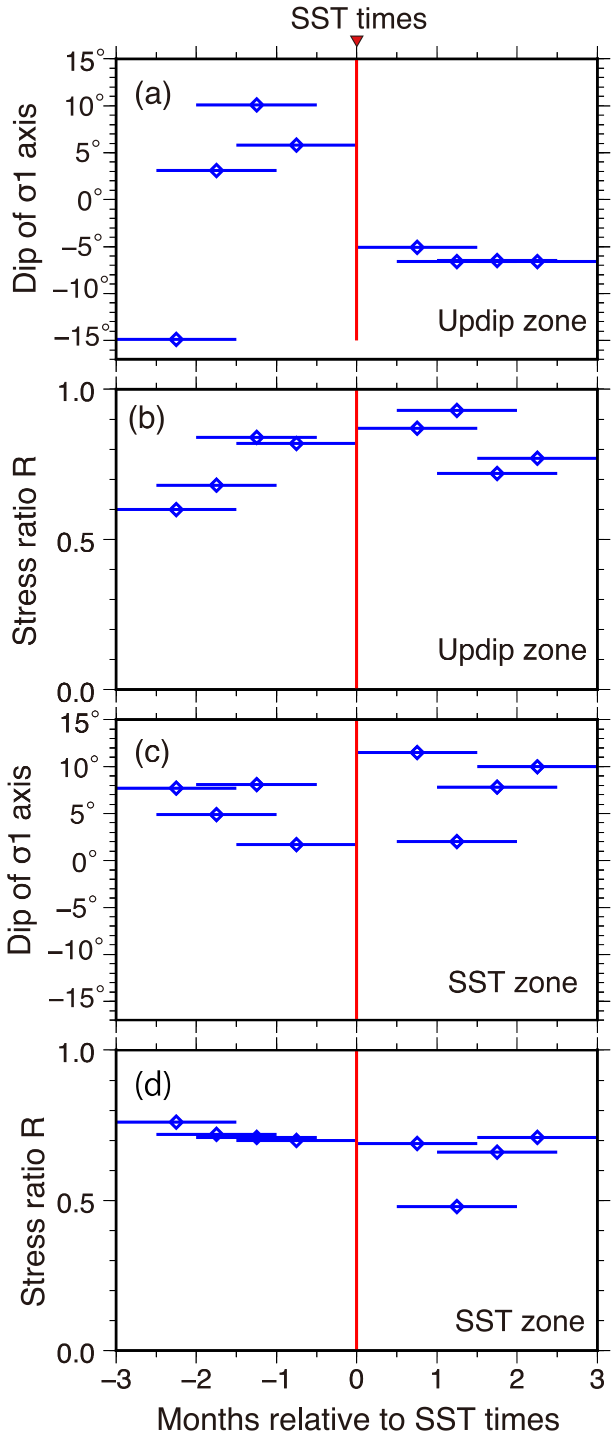

Supplementary Fig. 10.
Time evolution of dip of σ1 and stress ratio from the stress inversions. (a) dip of σ1 (the most compressive principal direction) for in-slab events in 1.5 mo windows in the region updip of SST (see Figure S8). (b) stress ratios for in-slab events in 1.5 mo windows in the region updip of SST (see Table S3). (c) as in (a) but for in-slab events below the SST region (see Fig. S9). (d) as in (b) but for in-slab events below the SST region (Table S4).

Supplementary Table 1.

Results of stress tensor inversions: Upper-plane events beneath the SST zone of the three regions (Region A+B+C), upper-plane events beneath the updip of the SST zone of the three regions, and oceanic mantle events (interplane and lower-plane events) beneath the three regions. σ1: maximum compressive stress, σ2: intermediate principal stress, σ3: minimum compressive stress, Misfit: average misfit angle. The contents of Supplementary Table 1 plotted on the lower hemisphere are also shown in Figs. 2e through 2j.

Event name	σ1				σ2				σ3				Misfit		Num. of events		Stress ratio R	
	Before		After		Before		After		Before		After		Before	After	Before	After	Before	After
	strike	dip	strike	dip	strike	dip	strike	dip	strike	dip	strike	dip						
Updip zone (Upper-plane)	335.5	5.9	163.3	7.03	87.5	74.5	35.9	78.5	244	14.2	254.5	9.0	20.1	12.3	23	34	0.83	0.88
SST zone (Upper-plane)	351.9	4.6	349.2	9.3	187.8	85.2	145.1	79.8	82.0	1.3	258.5	4.1	15.3	17.4	45	42	0.71	0.67
Oceanic mantle, entire (inter-plane and lower-plane)	299.1	38.1	355.2	80.3	92.2	48.7	110.4	4.17	198.2	13.6	201.1	8.7	32.9	44.8	67	59	0.31	0.09

Supplementary Table 2.

Results of stress tensor inversions: Interplane and lower-plane events (oceanic mantle events) beneath the SST zone, updip of the SST zone, and downdip of the SST zone of the three regions. The contents of Supplementary Table 2 plotted on the lower hemisphere are also shown in Supplementary Figs. 3a, 3b, 3e, 3f, 3i, and 3j.

Event name	σ1				σ2				σ3				Misfit		Num. of events		Stress ratio R	
	Before		After		Before		After		Before		After		Before	After	Before	After	Before	After
	strike	dip	strike	dip	strike	dip	strike	dip	strike	dip	strike	dip						
Oceanic mantle, updip zone	317.8	20.1	135.3	31.4	154.5	69.1	351.4	52.9	49.8	5.5	351.4	52.9	15.6	26.5	11	10	0.64	0.91
Oceanic mantle, SST zone	57.3	73.8	291.3	62.7	287.9	10.5	106.2	27.3	195.6	12.3	197.2	2.1	33.2	40.2	27	21	0.25	0.53
Oceanic mantle, downdip zone	286.0	25.5	35.3	78.1	67.7	58.7	284.7	4.2	187.7	16.9	193.8	11.1	22.4	35.7	29	28	0.51	0.13

Supplementary Table 3.
Results of stress tensor inversions: Upper-plane events beneath the updip of the SST zone of the three regions using a 1.5-month time window. The contents of Supplementary Table 3 plotted on the lower hemisphere are also shown in Supplementary Figs. 8a through 8c and 8g through S8i.

	Sigma 1		Sigma 2		Sigma 3				
Events name	strike	dip	strike	dip	strike	dip	misfit	Num. of events	Stress ratio R
-3.0 to -1.5 months	342.0	-14.9	19.3	71.5	254.9	10.7	25.3	40.0	0.60
-2.5 to -1.0 months	334.8	3.1	74.3	72.0	243.8	17.7	18.9	34.0	0.68
-2.0 to -0.5 months	331.9	10.1	92.0	70.6	238.9	16.5	17.0	27.0	0.84
-1.5 to 0.0 months	335.5	5.8	141.1	84.0	245.3	1.5	15.3	39.0	0.82
0 to 1.5 months	348.1	-5.1	64.9	68.8	260.0	20.5	11.4	36.0	0.87
0.5 to 2.0 months	343.8	-6.6	52.9	72.1	255.8	16.6	12.4	35.0	0.93
1.0 to 2.5 months	342.7	-6.5	37.9	78.7	253.8	9.2	17.2	32.0	0.72
1.5 to 3.0 months	340.9	-6.6	41.6	76.7	252.3	11.5	17.7	33.0	0.77

Supplementary Table 4.
Results of stress tensor inversions: Upper-plane events beneath the SST zone of the three regions using a 1.5-month time window. The contents of Supplementary Table 4 plotted on the lower hemisphere are also shown in Supplementary Figs. 9a through 9c and 9g through 9i.

	Sigma 1		Sigma 2		Sigma 3				
Events name	strike	dip	strike	dip	strike	dip	misfit	Num. of events	Stress ratio R
-3.0 to -1.5 months	349.7	7.7	132	80.3	258.9	5.9	13.4	40	0.76
-2.5 to -1.0 months	348.1	4.9	205	83.9	78.4	3.6	16.7	34	0.72
-2.0 to -0.5 months	351.6	8.1	204	80.4	82.4	5.1	14.5	27	0.71
-1.5 to 0.0 months	353.2	1.7	241	85.5	83.4	4.1	17.7	39	0.70
0 to 1.5 months	349.7	11.5	151	77.8	258.9	3.9	15.8	36	0.69
0.5 to 2.0 months	350.6	2.0	114	86.4	260.5	3.0	18.7	35	0.48
1.0 to 2.5 months	352.7	7.8	123	78.0	261.5	9.1	28.3	32	0.66
1.5 to 3.0 months	349.4	10.0	167	80.0	259.3	0.4	22.7	33	0.71
